# Skin testing versus radioallergosorbent testing for indoor allergens

**DOI:** 10.1186/1476-7961-3-4

**Published:** 2005-04-15

**Authors:** Birjis Chinoy, Edgar Yee, Sami L Bahna

**Affiliations:** 1 Allergy and Immunology Section, Louisiana State University Health Sciences Center; Shreveport, Louisiana, USA

**Keywords:** Allergy, Skin testing, RAST, Specific IgE, Mite, Cockroach, Cat, Dog

## Abstract

**Background:**

Skin testing (ST) is the most common screening method for allergy evaluation. Measurement of serum specific IgE is also commonly used, but less so by allergists than by other practitioners. The sensitivity and specificity of these testing methods may vary by type of causative allergen and type of allergic manifestation. We compared ST reactivity with serum specific IgE antibodies to common indoor allergens in patients with respiratory allergies.

**Methods:**

118 patients (3 mo-58 yr, mean 12 yr) with allergic rhinitis and/or bronchial asthma had percutaneous skin testing (PST) supplemented by intradermal testing (ID) with those allergens suspected by history but showed negative PST. The sera were tested blindly for specific IgE antibodies by the radioallergosorbent test (Phadebas RAST). The allergens were *D. farinae *(118), cockroach (60), cat epithelium (90), and dog epidermal (90). Test results were scored 0–4; ST ≥ 2 + and RAST ≥ 1 + were considered positive.

**Results:**

The two tests were in agreement (i.e., either both positive or both negative) in 52.2% (dog epidermal) to 62.2% (cat epithelium). When RAST was positive, ST was positive in 80% (dog epidermal) to 100% (cockroach mix). When ST was positive, RAST was positive in 16.3% (dog epidermal) to 50.0% *(D. farinae)*. When RAST was negative, ST was positive in 48.5% (cat epithelium) to 69.6% (*D. farinae*). When ST was negative, RAST was positive in 0% (cockroach) to 5.6% (cat epithelium). The scores of ST and RAST showed weak to moderate correlation (r = 0.24 to 0.54). Regardless of history of symptoms on exposure, ST was superior to RAST in detecting sensitization to cat epithelium and dog epidermal.

**Conclusion:**

For all four indoor allergens tested, ST was more sensitive than RAST. When both tests were positive, their scores showed poor correlation. Sensitizations to cat epithelium and dog epidermal are common, even in subjects who claimed no direct exposure.

## Background

Skin testing (ST) and specific serum IgE antibody measurement are commonly used in allergy evaluation. Percutaneous skin testing (PST) is the most common screening method. Intradermal testing (ID) is usually used for aeroallergens that show negative PST, yet are suspected by the patient or by the environmental history. ST requires the discontinuation of antihistamines and other drugs that have antihistaminic effect for intervals ranging from days to weeks before testing. Serum specific IgE measurement by the radioallergosorbent test (RAST) or its analogues is also frequently used, albeit more commonly so by non-allergists. In some situations, RAST may be preferred over ST [1]. In clinical practice, it is of importance to know the reliability of RAST compared to ST. Inhalation provocation testing would be the most reliable for respiratory allergies, but its clinical use in practice is limited to occupational cases. The objective of the present study was to compare ST with RAST for indoor aeroallergens in patients with respiratory allergies.

## Methods

### Patients

118 patients (ages 3 - 58 yr, mean 12 yr) with a history of respiratory allergies (allergic rhinitis and/or asthma) were routinely evaluated in the allergy clinic.

### Skin Testing

ST was done with extracts of the common aeroallergens. Commercial crude extracts (1:10 in 50% glycerin; Hollister-Stier, Spokane, WA) were used for PST (scratch method). Aeroallergens that showed negative PST in spite of a suggestive history were tested intradermally (ID) with 1:1000 crude aqueous extracts. Positive and negative controls were included using histamine (1 mg/ml for PST and 0.01 mg/ml for ID) and normal saline solution, respectively. The test result was read at 20 minutes for PST and at 15 minutes for ID testing. ST (PST and ID) was scored 0–4 as compared to the negative and positive controls [2], ST reactions ≥ 2 + were considered positive.

### Specific IgE

Sera from most patients were tested in a blind fashion for specific IgE antibodies by Phadebas RAST (Pharmacia Diagnostics, Kalamazoo, MI) and the result was scored 0–4 according to the manufacturer's criteria; scores ≥ 1 + (≥ 0.35 PRU/ml) were considered positive.

### Allergens

Four common indoor allergens were studied, namely: *Dermatophagoides farinae*, cockroach mix, cat epithelium, and dog epidermal.

### Statistics

Chi-square test was used for comparing frequencies (or percentages). Student's *t*-test was used for comparison of two means. Correlation coefficient was calculated for quantitative relationships.

## Results

**Figure 1:**
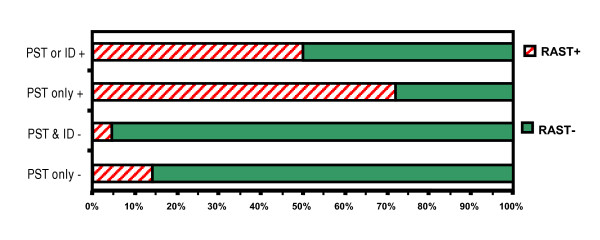
Comparison between skin testing & RAST for *D. farinae *in patients with respiratory allergy.

**Figure 2:**
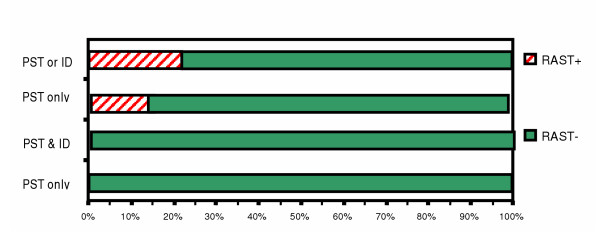
Comparison between skin testing & RAST for cockroach mix in patients with respiratory allergy.

**Figure 3:**
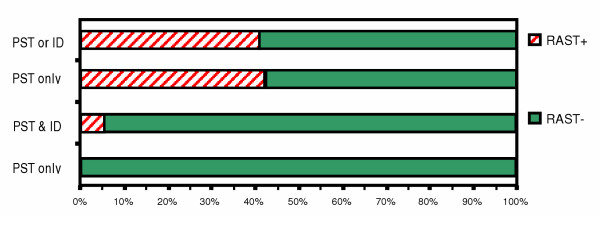
Comparison between skin testing & RAST for cat epithelium in patients with respiratory allergy.

**Figure 4:**
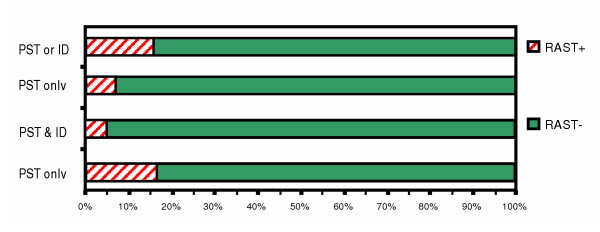
Comparison between skin testing & RAST for dog epidermal in patients with respiratory allergy.

The concordances and discordances of ST (PST ± ID) and RAST are presented in Table 1. The two tests were in agreement (i.e., both positive or both negative) in 52.2% (dog epidermal) to 62.2% (cat epithelium). When RAST was positive, ST was also positive in 80% (dog epidermal) to 100% (cockroach mix). When ST was positive, RAST was also positive in 16.3% (dog epidermal) to 50.0% *(D. farinae)*. When RAST was negative, ST was positive in 48.5% (cat epithelium) to 69.6% (*D. farinae*). When ST was negative, RAST was positive in 0% (cockroach) to 5.6% (cat epithelium). Comparisons of the RAST results with the results of PST and ID tests, separately or in combination, are presented in Figures 1, 2, 3, 4.

**Table 1:** Concordance and discordance between skin testing (PST ± ID) and RAST in all patients tested for *D. farinae*, cockroach mix, cat epithelium and dog epidermal.

**ST & RAST comparison**	***D. farinae *** n = 118	**Cockroach ** n = 60	**Cat epithelium ** n = 90	**Dog epidermal ** n = 90
**Concordance Both + or -**	69/118 (58.5%)	32/60 (53.3%)	56/90 (62.2%)	47/90 (52.2%)
**ST+ of RAST+**	48/49 (98.0%)	8/8 (100%)	22/24 (91.7%)	8/10 (80.0%)
**ST- of RAST+**	1/49 (2.0%)	0/8 (0%)	2/24 (8.3%)	2/10 (20.0%)
**ST+ of RAST-**	48/69 (69.6%)	28/52 (53.8%)	32/66 (48.5%)	41/80 (51.3%)
**ST- of RAST-**	21/69 (30.4%)	24/52 (46.2%)	34/66 (51.5%)	39/41 (48.7%)
**RAST+ of ST+**	48/96 (50.0%)	8/36 (22.2%)	22/54 (40.7%)	8/49 (16.3%)
**RAST- of ST+**	48/96 (50.0%)	28/36 (77.8%)	32/54 (59.3%)	41/49 (83.7%)
**RAST+ of ST-**	1/22 (4.5%)	0/24 (0%)	2/36 (5.6%)	2/41 (4.9%)
**RAST- of ST-**	21/22 (95.5%)	24/24 (100%)	34/36 (94.4%)	39/41 (95.1%)

For *D. farinae *(Fig. 1), when ST was positive by either PST or ID, RAST was positive in only 50.0%, whereas when PST and ID were both negative, RAST was negative in 95.5%. When only PST was positive, RAST was positive in 72%, whereas when PST was negative, RAST was negative in 86.0%.

For cockroach mix (Fig. 2), when ST was positive by either PST or ID, RAST was positive in only 22%, whereas when PST and ID were both negative, RAST was negative in 100%. When only PST was positive, RAST was positive in 15%, whereas when PST was negative, RAST was negative in 100%.

For cat epithelium (Fig. 3), when ST was positive by either PST or ID, RAST was positive in only 41%, whereas when PST and ID were both negative, RAST was negative in 94%. When only PST was positive, RAST was positive in 43%, whereas when PST was negative, RAST was negative in 0%.

For dog epidermal (Fig. 4), when ST was positive by either PST or ID, RAST was positive in only 16%, whereas when PST and ID were both negative, RAST was negative in 95%. When only PST was positive, RAST was positive in 7.0%, whereas when PST was negative, RAST was negative in 83%.

Regardless of history of symptoms on exposure, ST was superior to RAST in detecting sensitization to cat epithelium and dog epidermal (Table 2). In subjects who gave no history of significant exposure to cat or dog, sensitization was detected to cat epithelium in 45% by ST vs. 12% by RAST, and to dog epidermal in 36% by ST vs. 5% by RAST. In patients who had exposure to cat or dog, both ST and RAST tended to be more frequently positive when the patient was aware of symptoms on exposure. The positivity of ST or RAST to cat epithelium and dog epidermal did not differ much relevant to the patient's awareness of a cause-and-effect relationship.

**Table 2:** Skin test (PST+ID) and RAST positivity to cat epithelium and dog epidermal according to history of exposure & symptoms

**History of exposure & symptoms**	**Cat epithelium**		**Dog epidermal**	
	**ST+**	**RAST+**	**ST+**	**RAST+**
Symptoms on exposure	84%	47%	80%	27%
No symptoms on exposure	75%	45%	64%	11%
No history of exposure	45%	12%	36%	5%

The scores of ST (PST ± ID) and RAST (Table 3) for all patients generally showed weak to moderate correlations (r = 0.24 to 0.54). However, when the analysis was limited to patients in whom both tests were positive, there was a weak, non-significant correlation between the scores of the two tests (r = 0.04 to 0.37).

**Table 3:** Correlation coefficient (r) between Skin Test (PST ± ID) and RAST scores in patients with respiratory allergies

**Allergen**	**Patients tested**		**Patients positive by both ST & RAST**	
	**r**	**p**	**r**	**p**
*D. farinae*	0.54	<0.001	0.20	NS
Cockroach mix	0.42	<0.001	0.05	NS
Cat epithelium	0.48	<0.0001	0.04	NS
Dog epidermal	0.24	<0.05	0.37	NS

r: strong 0.8+, moderate 0.4 to <0.8, weak < 0.4

## Discussion

In the present study of patients with respiratory allergies, the ST and RAST results showed moderate concordance to the common indoor allergens studied (*D. farinae*, cockroach mix, cat epithelium and dog epidermal). The two tests were in agreement (either both positive or both negative) in 52.2% for dog epidermal to 62.2% for cat epithelium. Compared to RAST, ST was more commonly positive for all four allergens. When PST was positive, RAST was negative in 93% for dog epidermal, 85% for cockroach mix, 57% for cat epithelium and 28% for *D. farinae*. When ID was performed with the allergens that were negative by PST, the positivity of ST increased for all four allergens. When both the PST and ID tests were negative, RAST positivity did not exceed 6%. When both ST and RAST were positive, their scores showed weak non-significant correlations (r = 0.04 to 0.37).

Haahtela and Jaakonmäki [3] reported that in patients with positive ST to various allergens, RAST was positive in only 53%. Pascual et al [4] reported a positive ST and RAST in 55.6% for *D. farinae *and noted that RAST was negative in all patients who had a negative ST. Eriksson et al [5] reported a positive ST and RAST in 40% for dog dander and 73% for cat dander. They did not provide data on RAST positivity when ST was negative. In a study by Collins-Williams and Bremner [6], *D. farinae *RAST was negative in 6 who had positive ST, whereas RAST was positive in only 1 out of 41 patients with a negative ST. For cat hair, RAST was negative in 7 who had positive ST and was positive in none of 31 negative ST. For dog hair, RAST was negative in 12 who had positive ST and was positive in only 1 out of 31 whose ST was negative.

Tang and Wu [7] noted a strong concordance of 97% between ID testing and RAST for *D. farinae*, and ST was negative in 1 out of 30 patients with positive RAST. On the other hand, the concordance of ID testing and RAST for dog epidermal was 57%, and RAST was negative in 6 out of 23 positive ID tests. van der Zee et al [8] reported that *D. farinae *RAST was negative in 33 out of 281 (12%) patients with ID positive tests, and was positive in only 11 out of 379 (3%) with negative ID tests. For cat dander, RAST was negative in 45 out of 212 (21%) patients with positive ID tests, and was positive in only 2 out of 448 (0.4%) with negative ID tests. The poor correlation noted in our study between the scores of ST and RAST, even when both tests were positive, was also reported by Paggiaro et al [9].

The discrepancies between ST and RAST can be due to multiple factors. First, differences in the underlying immunologic basis of the two tests. ST is an *in vivo *biologic test that mimics the natural immediate-type hypersensitivity reaction, i.e., contact between the allergen and its specific IgE antibody on the mast cell, resulting in the local release of mediators and the formation of wheal-and-flare. On the other hand, RAST is an *in vitro *measurement of the level of circulating IgE antibodies in the serum, which may not reflect the tissue-fixed IgE antibodies. Second, differences in the allergenic quantity between the extracts used in ST and those used for in *vitro testing *[10]. When a purified and standardized *D. farinae *preparation was used for both ST and RAST, a high concordance of 84% was noted [11]. Nevertheless, RAST was negative in 8 out of 16 positive ST and was positive in only 1 out of 17 negative ST. Vanto et al [12] noted that the efficiency of RAST was increased by using dog dandruff instead of dog epithelium. Third, several studies reported marked variations in the efficiency of various *in vitro *assays for specific serum IgE antibodies [8,12-15], and of various ST techniques [16,17].

Both ST and RAST positivities to cat epithelium and dog epidermal were highest in patients who reported symptoms on exposure, followed by those who did not report such a relationship. The higher sensitivity of ST over RAST to cat epithelium and dog epidermal was noted regardless of the patient's awareness of causal relationship between symptoms and exposure. Interestingly, in patients who claimed no history of exposure to cat or dog, the ST was positive to cat epithelium in 45% and to dog epidermal in 36%. Such allergens are ubiquitous and have been noted in places where such animals do not exist, such as furniture stores [18] and schools [19].

It is of particular interest that RAST to cockroach was negative in 100% of cases that were negative to ST. To the best of our knowledge, there have been no relevant studies in the literature.

Finally, it is worth noting that our findings on specific IgE were based on using Phadebas RAST and should not be extrapolated to the more sensitive ImmunoCAP method (Pharmacia Diagnostics; Kalamazoo, MI) [1,20].

## Conclusion

Skin testing, particularly when PST was supplemented with ID, was more sensitive than Phadebas RAST in identification of the four indoor allergens we studied. However, RAST (or its analogues) would be indicated as a substitute for ST in certain cases [1,15] such as patients with dermographism, dermatitis, or who cannot discontinue antihistamines. It may also be preferred in patients with phobia to ST or in infants who have a few suspected allergens. It would be also safer than ST in patients with severe reactions to trivial exposures through inhalation or skin contact [21]. The high sensitization rate to cat and dog allergens in spite of the lack of direct exposure to such pets, underscores the high prevalence of such unsuspected, ubiquitous allergens.

## List of abbreviations

ST: skin testing

PST: percutaneous skin testing

ID: intradermal testing

RAST: radioallergosorbent test

## Competing interests

The author(s) declare that they have no competing interests.

## Authors' contributions

*Birjis Chinoy, MD*: data analysis, literature search, abstract presentation, manuscript preparation.

*Edgar Yee, MD*: study design, laboratory work, data gathering, data analysis.

*Sami Bahna, MD, DrPH*: planning, supervision and participation throughout the study and manuscript preparation
